# Phage Display Engineered T Cell Receptors as Tools for the Study of Tumor Peptide–MHC Interactions

**DOI:** 10.3389/fonc.2014.00378

**Published:** 2015-01-12

**Authors:** Geir Åge Løset, Gøril Berntzen, Terje Frigstad, Sylvie Pollmann, Kristin S. Gunnarsen, Inger Sandlie

**Affiliations:** ^1^Nextera AS, Oslo, Norway; ^2^Centre for Immune Regulation, Oslo University Hospital, University of Oslo, Oslo, Norway; ^3^Department of Biosciences, University of Oslo, Oslo, Norway

**Keywords:** phage display, tumor immunity, antigen presentation, T cell receptor, immunotherapy

## Abstract

Cancer immunotherapy has finally come of age, demonstrated by recent progress in strategies that engage the endogenous adaptive immune response in tumor killing. Occasionally, significant and durable tumor regression has been achieved. A giant leap forward was the demonstration that the pre-existing polyclonal T cell repertoire could be re-directed by use of cloned T cell receptors (TCRs), to obtain a defined tumor-specific pool of T cells. However, the procedure must be performed with caution to avoid deleterious cross-reactivity. Here, the use of engineered soluble TCRs may represent a safer, yet powerful, alternative. There is also a need for deeper understanding of the processes that underlie antigen presentation in disease and homeostasis, how tumor-specific peptides are generated, and how epitope spreading evolves during tumor development. Due to its plasticity, the pivotal interaction where a TCR engages a peptide/MHC (pMHC) also requires closer attention. For this purpose, phage display as a tool to evolve cloned TCRs represents an attractive avenue to generate suitable reagents allowing the study of defined pMHC presentation, TCR engagement, as well as for the discovery of novel therapeutic leads. Here, we highlight important aspects of the current status in this field.

## Introduction

T cells initiate and regulate adaptive immune responses to infections, are major components of allergic and autoimmune responses as well as transplant rejection, and play a pivotal role in cancer immune surveillance (1). The cancer-prone phenotypes of mice that lack components of the adaptive immune system strongly points to lymphocytes as critical factors in the anti-tumor activity (2). That the T cells represent the critical lymphocyte population is further underscored by a correlation between the presence of tumor infiltrating lymphocytes (TILs) and ability to control tumor growth. The CD45RO^+^ memory sub-group of the CD3 T cell compartment appears responsible of this activity (3, 4), and the CD8^+^ and CD4^+^ T cells probably act in concert (5). Furthermore, the observation that selective CD4^+^ T_H_ cell silencing may abrogate the anti-tumor response points to the CD4^+^ T_H_ cells as crucial (6, 7). It is also clear that adoptive cell therapy (ACT) through the use of CD8^+^ cytotoxic T lymphocyte (CTL) or CD4^+^ T_H_ cells may both result in durable anti-tumor activity (8–10). This is not merely a consequence of specific T cell target recognition, nor the affinity by which the T cell receptor (TCR) recognizes the target (11–13). Thus, to further delineate the mechanisms that lead to successful anti-tumor responses and how these can be exploited, it becomes imperative to further characterize the TCR–peptides bound to MHC molecules (pMHC) interaction, both at the cellular and the molecular level. The latter has posed a challenge to the field, since recombinant soluble TCRs have proven difficult to manufacture and work with. Consequently, our ability to study this pivotal interaction still depends on technology development (14). As such, protein engineering using combinatorial technologies is a powerful tool (15). Here, we focus on examples derived from the most prevalent combinatorial platform technology, namely phage display (16).

## T Cell Specificity at the Molecular Level

T cell function relies on productive binding between TCRs and antigens, which are proteolytically derived pMHC displayed on the surface of a variety of antigen presenting cells (APCs). Most TCRs bind pMHC ligands in a semi-conserved diagonal orientation with the somatically derived CDR3 loops located centrally atop the bound peptide, and the germ-line encoded variable CDR1 and CDR2 loops positioned over the MHC α helices (17). Upon activation, T cells may proliferate, differentiate, release cytokines, kill target cells, or carry out other effector functions. Thus, the ability of T cells to orchestrate the adaptive anti-tumor response depends on the TCR–pMHC interaction and downstream signaling events (18, 19). Productive interactions between TCRs and pMHCs are among the weakest known to initiate a biological response (20–22). Thus, a T cell needs to discriminate between foreign and self-peptides bound to MHC molecules even though the differences in affinity and binding kinetics may be minute (21, 22). Nonetheless, the earliest events in TCR signaling are characterized by high sensitivity and selectivity toward agonist pMHC (19). This is remarkable considering the apparent promiscuity of TCR binding, which in extreme cases have been suggested to be in the range of 10^6^ different peptides, yet still in a HLA restricted context (23). Such scaffold-dependent ligand binding promiscuity may partly be attributed to germ-line encoded HLA interaction signatures that ensure preservation of HLA restriction (24–27). It could also be an important feature explaining how a limited number of TCR germ line segments in combination with somatically generated CDR3 loops serve as versatile building blocks that generate a supply of TCRs able to promptly respond to a universe of pathogens (28–30). Clearly, multi-epitope specificity can also be a characteristic of tumor-specific TCRs, as shown in the study of Chinnasamy et al. focusing on HLA-A2/MAGE-A3 targeting (31). However, during ACT, such lack of mono-specificity may translate into fatal toxicity, underscoring the need for improved procedures for pre-clinical testing (32). Also, there is a need for a very precise delineation of how a TCR actually sees its cognate pMHC target, since minute structural changes may translate into very different cellular responses (33). Here, elucidating the underlying thermodynamic parameters governing the interaction may give clues to the rules that dictate TCR specificity (34, 35). Such biophysical insight may be further complemented by precise delineation of docking modes and binding studies that mimic the cellular topology (36, 37). In either case, one will need access to sufficient amounts of pure and stable soluble TCR and pMHC proteins.

## Reductionist Approach to Understanding the pMHC–TCR Interaction – The TCR Expression Problem

T cell receptors are membrane anchored proteins, and it is challenging to obtain sufficient quantities of recombinant soluble TCRs for molecular studies. A variety of approaches have therefore been adopted, including formation of single chain (sc) TCR, an analog to scFv antibody (Ab) fragments, and fusion of the extracellular TCR domains to other proteins; i.e., maltose binding protein, thioredoxin, human constant kappa domain, or leucine zippers (38–42). However, all of these strategies have had limited success due to low production yield and poor functionality. The most widely applied format as of today is the disulfide-bond linked TCRs (dsTCRs), which have a non-native disulfide bridge between the TCR constant domains (43). The method has significantly increased the stability and improved the folding characteristics of several human TCRs (44) when refolded from inclusion bodies, whereas direct soluble expression appears of limited utility (44, 45). An alternative approach is periplasmic expression with simultaneous over-expression of the chaperone FkpA, which has a huge impact on both the yield and functionality of the TCRs expressed (46). However, despite the optimized and improved methods, all are laborious and the expression levels vary extensively between individual clones. Thus, in many cases engineering of the TCR scaffold for higher stability, solubility and clone independent expression levels appears needed to obtain high quality protein.

## TCR Stability Can be Engineered by Use of Phage Display

**Figure 1:**
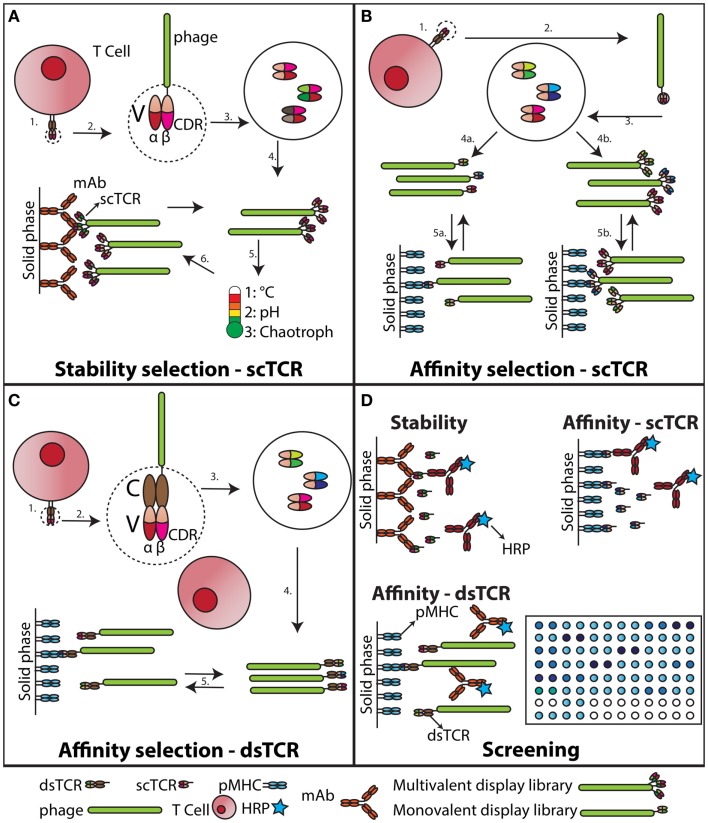
**Stability engineering and affinity maturation of soluble TCRs**. **(A)** Stability engineering of scTCR. The variable (V) regions of an individual TCR (1) are cloned and connected via a synthetic linker (55). This scTCR is expressed as fusion to the M13 pIII capsid protein (2). The scTCR is then diversified by *in vitro* mutagenesis (3). This collection of mutagenized scTCRs are expressed as a high valence display phage library (4), which is challenged with increased temperature, unfavorable acid/base, or chaotropic conditions (5). Stabilized scTCR resisting aggregation despite the challenge is retrieved by capture on a conformation-specific ligand, such as an Ab (6). **(B)** Affinity maturation of scTCR. The V regions of an individual TCR (1) are cloned and expressed as a scTCR fusion to either the M13 pIII (55), or pIX capsid protein (56) (2). Individual TCR α- and β-chain CDR loops of the scTCR are randomized to create diversity (3). This collection of mutagenized scTCRs is then expressed as a low (4a) or high (4b) valence display phage library, which is selected against pMHC (5a and b). **(C)** Affinity maturation of dsTCR. The V regions of an individual TCR (1) are cloned and expressed as fusions to prototypic constant (C) domains stabilized by an artificial disulfide bridge, hence reconstituting the complete TCR ectodomain architecture (43). This recombinant dsTCR is then expressed as fusion to the M13 pIII capsid protein (2). Individual TCR α- and β-chain CDR loops of the dsTCR are randomized to create diversity (3). Usually this process is confined to the *in vivo* pMHC specificity-determining CDR3 loops (57), but has also been successfully applied to the germ-line encoded CDR2 only (58, 59). This collection of mutagenized dsTCRs is then expressed as a low valence display phage library (57), which is selected against pMHC (5). **(D)** Screening of engineered dsTCR and scTCR. The stability engineered **(A)**, or affinity matured **(B)** scTCR is reformatted to soluble, periplasmic expression (46), and individual mutated scTCRs screened for functionality against target immobilized on solid phase. The screening for desired binders following dsTCR selection is done on phage due to incompatibility with high-throughput soluble dsTCR screening (45).

Evolution of recombinant proteins by random mutagenesis and subsequent *in vitro* selection has been successfully applied to a wide range of protein classes (47), and in particular antibodies (48). One such strategy has utilized selection of mutated heavy chain variable domains in combination with thermal challenge to obtain aggregation-resistant domains (49). Recently, guided by the study of Jespers et al., molecular evolution of a TCR for increased stability and expression was carried out by use of phage display (50). Libraries of randomly mutated scTCRs were produced as fusion to protein III on the surface of M13 phage. High valence display allowed stress-induced aggregation after thermal challenge (Figure 1). Variants characterized by markedly increased soluble expression levels and reduced aggregation propensity were obtained after rapid heating and cooling, followed by capture of aggregation-resistant scTCRs (Figures 1A,D). Importantly, over-expression of the periplasmic chaperone FkpA resulted in even display levels among the TCR library members, which proved imperative for successful selection. Thus, the previously identified folding assistance to soluble and phage displayed scTCRs offered by FkpA now allows for extended engineering opportunities to TCRs in conjunction with high-throughput soluble screening (Figures 1A,B,D). The list of strategies used for engineering of increased protein biophysical stability employing destabilization challenges in combination with multivalent phage display selection has been further extended. Famm et al. reported selection of Ig domains resistant to e.g., acidic pH induced aggregation with increased thermodynamic stability (51, 52). Furthermore, Christ et al. have reported a method for generation of Ab sub repertoires, based on combinatorial assembly of CDRs from an aggregation-resistant repertoire (53). Repeated cycles of selection and thermal denaturation generated domains with remarkable aggregation-resistant properties. Similar strategies may well be employed to obtain soluble TCR scaffolds with even higher expression levels and increased stability than reported to date (50, 54).

## TCR Affinity Can be Engineered by Use of Phage Display

To overcome the intrinsically low binding affinity of the TCR–pMHC interaction, two approaches have been utilized, namely multimerization and affinity maturation. Tetrameric forms of soluble TCRs have been produced by capturing biotinylated TCRs onto avidin, which have four binding sites for biotin (60, 61). The overall increased avidity greatly increases the half-life of the TCR–pMHC interaction. Such reagents are used in cellular binding assays, as they stably adhere to the cell surface. Crucial information may be collected that allows for deduction of biologically relevant information (61). However, for example direct assessment of peptide presentation at stoichiometric levels requires stronger binding between the TCR and pMHC than what is possible to reach with native TCRs (60). Therefore, affinity maturation of TCRs for increased binding has been performed. Again, phage display technology has been efficient (15, 57), and selection from mutant TCR display libraries can yield TCRs with dramatically increased affinities toward the cognate pMHCs without concomitant increase in cross-reactivity (Figures 1B–D). Crystallographic data show that this can be explained by a loss of flexibility in the otherwise entropically unfavorable TCR–pMHC interaction interphase, as well as an overall increase in shape complementarity (62). The degenerate pMHC interaction mode of TCRs could suggest that engineering must be restricted to the somatically derived CDR3 loops to preserve MHC restriction (26). However, this appears not to be the case as also the germ-line encoded CDR2 loop has been targeted by mutagenesis resulting in increased affinity (58, 63). Such engineered high-affinity TCRs have been used to study low level tumor associated pMHC presentation at physiological levels to obtain information that has previously been unattainable (64–66).

## Lessons Learnt – Translation to the Tumor pMHC Complexes and Cancer Therapy

Conformational plasticity in the CDR loops upon pMHC binding appears to be a driving mechanism upon TCR–pMHC complex formation, whereas, rigid “lock and key” interaction modes also have been reported (67). This energetic diversity reflects the multiple binding strategies implemented by the TCR during pMHC engagement. However, in spite of the described diversity (68), step by step, we are unveiling the mechanism by which a TCR deciphers a pMHC complex.

The low level of molecular shape complementarity in the TCR–pMHC complex gives rich opportunities for *in vitro* affinity maturation (57, 58, 62). This feature is likely to be generic to most TCRs (26). In the case of the affinity maturation of a HLA-A2/MART-1 specific TCR, the increase in shape complementarity was focused primarily onto the MHC portion of the complex essentially without affecting the peptide interaction (69). Thus, loss of peptide specificity could potentially be expected. However, structural and thermodynamic investigations suggested that this was not the case. In stark contrast, the opposite pMHC interaction strategy was employed by a different TCR recently reported, which was evolved toward high affinity against the HLA-A2/Tax complex. Here, a peptide-focused mechanism was found to underlie the enhanced affinity (59). Thus, the authors suggest an alternative interaction mode to the generally accepted two-step TCR–pMHC binding model (19). Here, instead of first docking the CDR1 and CDR2 onto the MHC, followed by kinetic proofreading of the peptide by the CDR3s, the opposite order of interaction is suggested. This scan-clamp model fits well with how weak, but specific protein–protein interactions have been stabilized by affinity clamping in other trimeric complexes analogous to TCR–pMHC (70, 71). It also explains how exquisite peptide specificity can be preserved both in natural and engineered systems. In either case, the picture is not consistent, and the observation that complementary structural fluctuations of both the antigenic peptide and the CDR3s of the TCR prevail even after final complex formation, underscores the remarkable flexibility of the interaction (35). Thus, at present it is challenging to validate both naturally and artificially evolved TCRs e.g., for safe use in therapy. Despite rigorous classical pre-clinical validation, a human clinical pilot study resulted in fatal cardiac toxicity due to unforeseen cross-reactivity when an affinity matured TCR against HLA-A1/MAGE-A3 was employed in specificity redirected ACT (32).

Also, there are still many questions to be answered regarding the difference between MHC class I and class II restricted TCRs. In particular, it is important to understand the significance of co-receptors in creating a fully functional immunological synapse (72). For instance, it has been shown that CD8 plays a stabilizing role in the TCR–pMHC class I interaction (73), whereas, CD4 does not appear to play a role in the corresponding TCR–pMHC class II interaction (36). Notably, these two co-receptor interactions differ significantly in MHC binding strength, which may possibly elude to their differential importance (72). Thus, an affinity threshold has been observed for the CD8 T cell compartment that limits the benefits of very high intrinsic affinity between TCR–pMHC class I (11, 74–77). So far, this has not been observed for TCR–pMHC class II. Even though fewer examples have been reported with respect to TCRs reactive toward pMHC class II, it appears that different functional rules govern this interaction (74, 77, 78), and pilot trials have shown promising results in pMHC class II restricted ACT (10, 79).

## Concluding Remarks

The ability to engineer stable and high-affinity TCRs offers a unique ability to harness the immune system with an improved ability to respond to a given pMHC. However, our current understanding is still incomplete with respect to how this can safely be translated into durable cancer immunotherapy (9). One would expect improved affinity to translate into improved killing ability, but the empirical data suggest otherwise. Rather, an affinity threshold limiting any additional benefit in cellular responses above a certain TCR–pMHC binding strength has been reported, as outlined above. Moreover, the affinity threshold appears to be largely confined to the CD8 T cell compartment, as nearly all high-affinity engineered CD4 T cells have responded with both improved peptide sensitivity and preserved specificity. This gives clues as to how one might differentially exploit TCRs derived from the two distinct T cell compartments. On one hand, engineered high-affinity MHC class I and II restricted TCRs may both serve as very potent cytotoxic drugs in a soluble format (80). On the other hand, the most potent avenue for redirected cell therapy might in some cases be limited to the MHC class II restricted compartment due to the CD8 T cell affinity threshold (10, 74).

A final question is whether or not one actually needs to apply ACT to achieve optimal clinical benefit. ACT is demanding as it relies on massive *ex vivo* autologous cell expansion, which will be difficult in major patient groups for example due to cellular senescence (81–83). Epitope spreading appears to be the signature of successful anti-tumor immune responses (10, 13, 84, 85). Now it appears that this can also be achieved by the use of soluble TCRs harnessed with the ability to recruit the endogenous adaptive effector apparatus (80, 86). Such soluble TCRs appear attractive compared to the cellular approaches in light of patient convenience and safety issues (32). The use of a soluble TCR obviates the need for *ex vivo* cell expansion and a single drug may be used by a genetically heterogeneous patient population sharing the target MHC allele only. Putative off target toxicity may also be better controlled, and quenched if needed, due to tunable dosing and limited drug half-life. A soluble drug would also be less prone to efficacy variation due to *in vivo* regulatory mechanisms than ACT. How well the soluble TCR approach is reduced to clinical practice is currently under investigation through a first in man phase I/II clinical trial in late-stage malignant melanoma targeting a HLA-A2/gp100 complex (http://www.clinicaltrials.gov/ and IMCgp100).

Undoubtedly, improved phage display technology will continue to be a driver in providing engineered TCRs, which will be powerful tools to monitor and elucidate specific pMHC complexes, as well as creating novel specificities suitable for safe use in the clinic.

## Conflict of Interest Statement

The authors declare that the research was conducted in the absence of any commercial or financial relationships that could be construed as a potential conflict of interest.
